# Photocatalytic Inactivation of Plant Pathogenic Bacteria Using TiO_2_ Nanoparticles Prepared Hydrothermally

**DOI:** 10.3390/nano10091730

**Published:** 2020-08-31

**Authors:** László Kőrösi, Botond Pertics, György Schneider, Balázs Bognár, János Kovács, Vera Meynen, Alice Scarpellini, Lea Pasquale, Mirko Prato

**Affiliations:** 1 Research Institute for Viticulture and Oenology, University of Pécs, Pázmány P. u. 4, H-7634 Pécs, Hungary; 2 Department of Medical Microbiology and Immunology, Medical School, University of Pécs, Szigeti st. 12, H-7624 Pécs, Hungary; pertics.botond@pte.hu (B.P.); schneider.gyorgy@pte.hu (G.S.); 3 Institute of Organic and Medicinal Chemistry, University of Pécs, Szigeti st. 12, H-7624 Pécs, Hungary; balazs.bognar@aok.pte.hu; 4 Environmental Analytical and Geoanalytical Research Group, Szentágothai Research Centre, University of Pécs, Ifjúság u. 20, H-7624 Pécs, Hungary; jones@gamma.ttk.pte.hu; 5 Laboratory of Adsorption and Catalysis, Department of Chemistry, University of Antwerp, Universiteitsplein 1, 2610 Wilrijk, Belgium; vera.meynen@uantwerpen.be; 6 Electron Microscopy Facility, Istituto Italiano di Tecnologia, via Morego 30, 16163 Genova, Italy; alice.scarpellini@iit.it; 7 Materials Characterization Facility, Istituto Italiano di Tecnologia, via Morego 30, 16163 Genova, Italy; Lea.Pasquale@iit.it (L.P.); Mirko.Prato@iit.it (M.P.)

**Keywords:** anatase, TiO_2_ nanoparticles, photocatalysis, antimicrobial nanomaterials, plant pathogens, reactive oxygen species, hydroxyl radicals, superoxide radicals, agriculture, nano-pesticide

## Abstract

Exploitation of engineered nanomaterials with unique properties has been dynamically growing in numerous fields, including the agricultural sector. Due to the increasing resistance of phytopathogenic microbes, human control over various plant pathogens in crop production is a big challenge and requires the development of novel antimicrobial materials. Photocatalytic active nanomaterials could offer an alternative solution to suppress the plant pathogens. In this work, titanium dioxide nanoparticles (TiO_2_ NPs) with high photocatalytic activity were synthesized by hydrothermal post-treatment of amorphous titania at different temperatures (250 °C or 310 °C) without using any additives or doping agents. The obtained samples were investigated through X-ray diffraction, N_2_-sorption measurements, diffuse reflectance UV-Vis spectroscopy, transmission electron microscopy, electron paramagnetic resonance spectroscopy, and X-ray photoelectron spectroscopy. The applied hydrothermal treatment led to the formation of TiO_2_ nanocrystallites with a predominant anatase crystal phase, with increasing crystallinity and crystallite size by prolonging treatment time. The photocatalytic activity of the TiO_2_ NPs was tested for the photo-degradation of phenol and applied for the inactivation of various plant pathogens such as *Erwinia amylovora*, *Xanthomonas arboricola* pv. *juglandis*, *Pseudomonas syringae* pv. *tomato* and *Allorhizobium vitis*. The studied bacteria showed different susceptibilities; their living cell numbers were quickly and remarkably reduced by UV-A-irradiated TiO_2_ NPs. The effectiveness of the most active sample prepared at 310 °C was much higher than that of commercial P25 TiO_2_. We found that fine-tuning of the structural properties by modulating the time and temperature of the hydrothermal treatment influenced the photocatalytic properties of the TiO_2_ NPs considerably. This work provides valuable information to the development of TiO_2_-based antimicrobial photocatalysts.

## 1. Introduction

Every year at least 20–40% of losses in crop yield are caused by pathogenic infections, which results in several billion dollars’ losses worldwide [1,2]. In the agricultural sector, the use of antibiotics is strictly limited in the control of bacterial diseases in several European countries and the USA [3]. The development of novel alternative antimicrobial solutions is therefore required, to fight against the bacterial pathogens and overcome the requirement to decrease the usage of conventional antibiotics in the plant/crop production. The use of nanomaterials with antimicrobial properties offers a potential solution for prevention. Among the various nanostructured materials with different properties, titanium dioxide (TiO_2_) is well-known for its particularly high photoreactivity [4]. The exploitation of this special characteristic represents a huge opportunity for both environmental and plant protection [5]. Photo-catalytically active TiO_2_ nanoparticles (NPs) are still being intensively studied for the degradation of toxic compounds, as indicated by the thousands of published papers. However, studies on the inactivation of plant pathogens with photocatalytic materials are much less available. 

Several studies have addressed the possibility of photocatalytic inactivation of phytopathogenic fungi using metal oxide semiconductors such as TiO_2_ [5], ZnO NPs [6], and titanate-silver nanocomposite [7]. Although ZnO exhibits photocatalytic activity, this oxide is easily soluble both in dilute acids or alkaline solutions due to its low chemical stability. Therefore, ZnO is not an ideal photocatalyst to be used in agricultural applications. Furthermore, ZnO shows an antifungal (fungistatic) effect even without light activation [8]. Thus, the biocidal action of ZnO is complex and not only related to its photocatalytic activity. *Botrytis cinerea* (*B. cinerea*) isolated from tomato has been inactivated with silver-functionalized hydrogen titanate nanotubes [7]. Trititanate (H_3_Ti_3_O_7_) is chemically more stable than ZnO; however, it has very low photocatalytic activity [9], which makes H_3_Ti_3_O_7_ nanotubes rather an adsorbent than a photocatalyst [10]. However, the decoration of titanate with biocidal silver nanoparticles led to an effective antimicrobial composite, which could be used to inactivate *B. cinerea* within 20 min [7]. TiO_2_, with its well-known polymorphs (anatase, rutile, brookite, and TiO_2_(B)), is a chemically very stable semiconducting metal oxide. Its pure forms have not been studied frequently in the photocatalytic inactivation of plant pathogens. Maneerat et al. studied the antifungal activity of TiO_2_ in the photocatalytic reaction [11]. They found both TiO_2_ powder and TiO_2_-coated films exhibited antifungal activity to control fruit rot. Sichel and co-workers found that TiO_2_ showed a strong tendency to attach to the fungus spore surface, which enhances the effectiveness of the photocatalytic disinfection [12]. For the photocatalytic inactivation of *Enterobacter cloacae* SM1 and *Erwinia carotovora subsp. carotovora* ZL1*, * sol-gel-based TiO_2_ films were used on a glass substrate [13]. More studies have been reported on modified or doped TiO_2_. For example, palladium-modified nitrogen-doped titanium oxide [14], nitrogen and fluorine co-doped TiO_2_ [15], copper-modified Ti^3+^ self-doped TiO_2_ [16] and TiO_2_/Cu_2_(OH)_2_CO_3_ nanocomposite [17] were prepared and tested against various pathogen fungi such as *Fusarium graminearum*, *Colletotrichum gloeosporioides*, *Botryosphaeria dothidea*, *Fusarium moniliforme*, and *Fusarium oxysporum f.* sp. *vasinfectum*. Furthermore, it should be noted that various doping agents (e.g., copper-based compounds) show fungicide effect which strengthens the antimicrobial effect of the photocatalyst. Besides pathogenic fungi, the photocatalytic killing of phytopathogenic bacteria has rarely been examined, and therefore only very limited relevant information is available in the literature.

In this work, we investigated the photocatalytic inactivation of four plant pathogenic bacteria with non-doped nanocrystalline TiO_2_ samples. We present a facile low-temperature hydrothermal synthesis for the preparation of TiO_2_ NPs. The effects of the synthesis conditions on the crystallinity, crystallite size, Brunauer–Emmett–Teller (BET) surface area, chemical composition, and the photocatalytic activity of TiO_2_ NPs were investigated in detail. Furthermore, we examined the formation kinetics of the main reactive oxygen species (ROS) during the photocatalytic reaction by electron paramagnetic resonance (EPR) spectroscopy. Relationships between physicochemical properties, ROS production ability, and photocatalytic activity of TiO_2_ NPs were evaluated.

## 2. Materials and Methods 

### 2.1. Materials

The following were used as received, 2-propanol (HiPerSolv CHROMANORM for HPLC, VWR, Radnor, PA, USA); titanium(IV) isopropoxide (≥97%, Sigma-Aldrich, St. Louis, MO, USA); Aeroxide TiO_2_ P25 (Degussa AG, Hanau-Wolfgang, Germany); phenol (≥99.0%, Sigma-Aldrich, St. Louis, MO, USA); and dimethyl sulfoxide (DMSO) (≥99.9%, Sigma-Aldrich, St. Louis, MO, USA). As previously described, 5,5-dimethyl-1-pyrroline *N*-oxide (DMPO), was synthesized [18], and it was freshly distilled before use. High purity deionized water was obtained by a LaboStar 7 TWF-UV (SG Wasseraufbereitung und Regenerierstation GmbH, Barsbüttel, Germany) system.

### 2.2. Bacterial Strains

For the pathogen inactivation study, four Gram-negative bacterium species such as the ATCC 49946 strain *Erwinia amylovora* (American Type Culture Collection, Manassas, VA, USA), the DSMZ 1049 strain *Xanthomonas arboricola* pv. *juglandis* (DSMZ-German Collection of Microorganisms and Cell Cultures, Braunschweig, Germany), the DC3000 strain *Pseudomonas syringae* pv. *tomato* and the *Allorhizobium vitis* isolates were used. Matrix-assisted laser desorption/ionization-time of flight mass spectrometry (MALDI-TOF MS) (Vitek MS, Biomerieux, Marcy-l’Étoile, France) was used to confirm the identity of individual bacterial colonies from fresh subcultures [19].

### 2.3. Synthesis

Whilst stirring, 7.4 mL of titanium(IV) isopropoxide was mixed with 15 mL of 2-propanol, and then 12.5 mL of deionized water was added dropwise to the mixture. The resulting white dispersion was centrifuged, and the sediment obtained was thoroughly washed with water. After the washing procedure, the sediment was redispersed in water and treated hydrothermally at 250 or 310 °C for 12, 24, 48, or 72 h. Finally, the sediments were dried at 50 °C for 12 h. The samples prepared at 250 °C were denoted A12h, A24h, A48h, and A72h, reflecting the duration of the hydrothermal treatment. The sample prepared at 310 °C for 24 h was denoted A310C.

### 2.4. Methods

Powder X-ray diffraction (XRD) patterns were acquired through the use of Cu Kα radiation with a Rigaku MiniFlex 600 X-ray diffractometer (Rigaku, Tokyo, Japan) operating at 40 kV and 15 mA. Crystallinity of the samples was determined as described earlier, by using ZnO as internal calibration standard [20]. Specific surface area and porosity measurements were carried out by nitrogen physisorption techniques at 77 K in a model Quantachrome QuadraSorb SI instrument (Quantachrome GmbH & Co. KG, Odelzhausen, Germany). Prior to the measurements, samples were degassed for 16 h at 200 °C under vacuum to remove weakly adsorbed species. The specific surface area values were evaluated using the multi-point BET (Brunauer–Emmett–Teller) model, considering 11 equally spaced points in the p/p_0_ range from 0.05 to 0.30. Bright-field transmission electron microscopy (BF-TEM) images have been acquired on a JEOL JEM-1011 instrument (JEOL Ltd., Tokyo, Japan) equipped with a thermionic W electron source and operated at an acceleration voltage of 100 kV.

UV-Vis diffuse reflectance measurements were performed on a thermo-Electron Evolution 500 double-beam spectrometer (Thermo Electron, Cambridge, UK) with an RSA-UC-40 diffuse reflectance accessory. Spectra are the average of 3 measurements of a 2% diluted sample in KBr. KBr was taken as background.

X-ray photoelectron spectroscopy (XPS) measurements were performed on a Kratos Axis Ultra^DLD^ spectrometer (Kratos Analytical, Manchester, UK) equipped with a monochromatic Al Kα source (20 mA, 15 kV). High-resolution narrow scans were acquired on Ti 2p and O 1s core levels at constant pass energy of 10 eV in steps of 0.1 eV. The photoelectrons were detected at a take-off angle of *Φ* = 0° to the surface normal. Spectra were analyzed using *CasaXPS* software, version 2.3.19, Casa Software Ltd. The binding energy (BE) scale was calibrated with the C 1s peak at 285 eV for the adventitious carbon. 

Electron paramagnetic resonance (EPR) spectra were recorded on a Miniscope MS200 spectrometer (Magnettech GMBH, Berlin, Germany) working with a modulation amplitude of 0.2 mT and a microwave power of 10.0 mW. Measurements were carried out in water (for OH^●^) or DMSO (for O_2_^●−^) at room temperature, containing 0.1 mg/mL of TiO_2_ NPs and 100 mM of freshly prepared DMPO. EPR spectra were recorded on the liquid obtained after 30, 60, 90, 120, 180, and 300 s of photoirradiation of TiO_2_ dispersions containing DMPO. For quantification of DMPO-adducts, 10 μM 3-(hydroxymethyl)-2,2,5,5-tetramethyl-2,5-dihydro-1H-pyrrol-1-oxyl was used as external standard. All the measurements were repeated three times. In the absence of illumination or TiO_2_ NPs, the EPR spectra of DMPO-adducts were not observed within the used time range. 

The phenol concentration was determined by high-performance liquid chromatography (HPLC). The measurements were performed on a PerkinElmer Series 200 HPLC system (PerkinElmer, Shelton, CT, USA), consisting of a vacuum degassing unit, quaternary pump, autosampler, column thermostat, and a diode-array detector (DAD). Chromatographic separations were achieved by using a Phenomenex Kinetex 2.6 μm XB-C18 100 Å, 100 × 4.6 mm column (Phenomenex, Torrance, CA, USA). The column temperature was maintained at 25 °C. Isocratic elution was applied. The mobile phase was a mixture of acetonitrile and water (30:70 *v*/*v*%) with a flow rate of 1 mL min^−1^. The injection volume was 5 μL, and the absorbance was monitored at 210 nm. A calibration curve for the quantification was obtained by measuring phenol solutions with known concentrations in the range 0.05−0.5 mM.

### 2.5. Photocatalytic Tests

The photocatalytic activity of TiO_2_ NPs was tested by the degradation of phenol as a model compound. For the photocatalytic tests, 50 mL of 0.5 mg/L aqueous TiO_2_ dispersions containing 0.5 mM phenol were used. Prior to the light exposure, the dispersions were stirred in the dark for 30 min. Subsequently, the dispersions were irradiated at room temperature for different durations by using a 15-W UV-A light source (Sylvania F15W/T8/BL368) (Feilo Sylvania Germany GmbH, Erlangen, Germany). The distance between the dispersion and lamp was 5 cm. Before analysis, the dispersions were centrifuged at 20,660× *g* for 10 min. The obtained supernatants were analyzed for phenol by HPLC-DAD. Commercial Degussa P25 was used as a reference photocatalyst.

### 2.6. Antibacterial Tests 

For antibacterial tests, we chose the A310C sample that showed the highest photocatalytic activity in the phenol photo-degradation, as well as Commercial Degussa P25 TiO_2,_ used as a reference photocatalyst. Tests with four bacterial species were performed as previously described with slight modifications [21]. Briefly, 50 μL of late logarithmic cultures of each microorganism were used as starter cultures and transferred into 50 mL of Luria broth (LB). Flasks were incubated in a shaking thermostat at 30 °C (120 rpm) till the optical density at 600 nm (OD_600_) reached 0.5. Cells were collected with centrifugation at 10,000× *g* for 10 min, then washed twice with 0.9 w/v% NaCl solution. Finally, optical densities were set to 1 (~1 × 10^8^ colony forming unit (CFU)/mL). Following that, 100 μL of these bacterial suspensions were transferred into 100 mL beakers, already containing 9.9 mL of 0.5 mg/mL TiO_2_ suspensions. These mixtures were stirred on a parallel magnetic stirrer platform installed in a closed dark box. After 15 min of dark incubation, the photocatalytic reactions were started by switching on a 15-W UV-A lamp (F15W/T8/BL368 fluorescent lamp, Sylvania) (Feilo Sylvania Germany GmbH, Erlangen, Germany). For bacterial enumeration, 10 µL of sample aliquots were taken at 0, 10, 20, and 30 min. The numbers of viable cells were determined by oozing 10 µL of suitably diluted aliquots onto LB agar plates and then counting the colonies after 48 h of incubation at 30 °C. All antibacterial tests were performed in three replicates.

## 3. Results

### 3.1. Structural Properties: Crystalline Structure, Specific Surface Area

**Figure 1:**
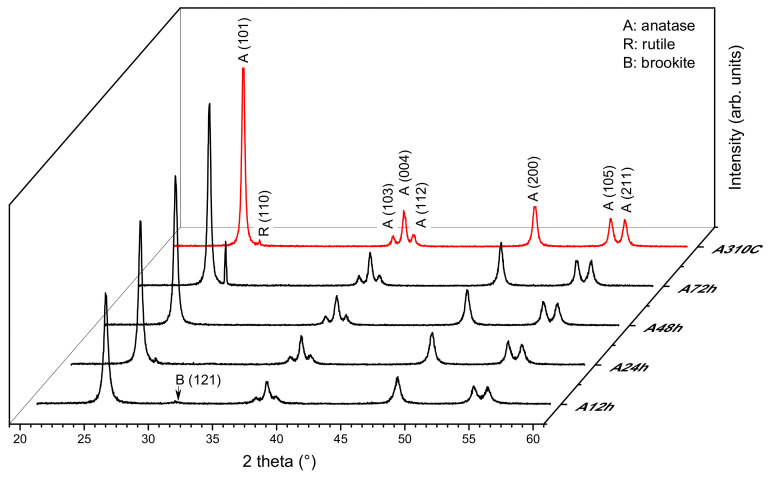
X-ray diffraction (XRD) patterns of TiO_2_ nanoparticles (NPs) treated hydrothermally at 250 °C for 12 h, 24 h, 48 h, and 72 h. A310C (red line) was prepared at 310 °C for 24 h. Characteristic XRD peaks of anatase (A), rutile (R), or brookite (B) are indexed.

XRD patterns of TiO_2_ NPs treated hydrothermally at 250 and 310 °C for different duration are displayed in Figure 1. Distinct diffraction peaks can be seen at 2*θ* = 25.4, 37.1, 38.0, 38.7, 48.1, 54.0, and 55.2° which are assigned to (101), (103), (004), (112), (200), (105), and (211) lattice planes of anatase (JCPDS no. 71-116), respectively. Although anatase was the predominant form for all the samples, rutile TiO_2_ (JCPDS no. 21-1276) with a characteristic peak at 2*θ* = 26.7° (assigned to the (110) plane) was also detected in the A24h, A72h, and A310C samples. Moreover, a very low intensity diffraction peak at 30.75° shows brookite traces (JCPDS card no. 29-1360) in A12h. The three crystal phases were quantified, and the obtained results are listed in Table 1.

Increasing the treatment time from 12 h to 72 h, hydrothermal treatment at 250 °C gradually increased the crystallinity of the samples from ~75 to ~100%. At the same time, the prolonged synthesis induced the formation of the rutile phase (7.6% at 72 h). At 310 °C, high crystallinity (~95%) was achieved in a three-times shorter treatment time (24 h). In this sample (A310C), the rutile phase did not exceed 1%.

Besides the crystallinity, the average crystallite size (*d*) was also changed by the treatment. In Figure 1, the full width at half maximum decreased with increasing treatment time, showing that hydrothermal treatment induced the growth of the crystalline domains. After the treatment at 250 °C, the average crystallite sizes of anatase NPs were 29.7 and 48.7 nm for A12h and A72h samples, respectively (Table 1). Anatase crystallites with sizes of ~52 nm were formed after 24 h at 310 °C.

**Figure 2:**
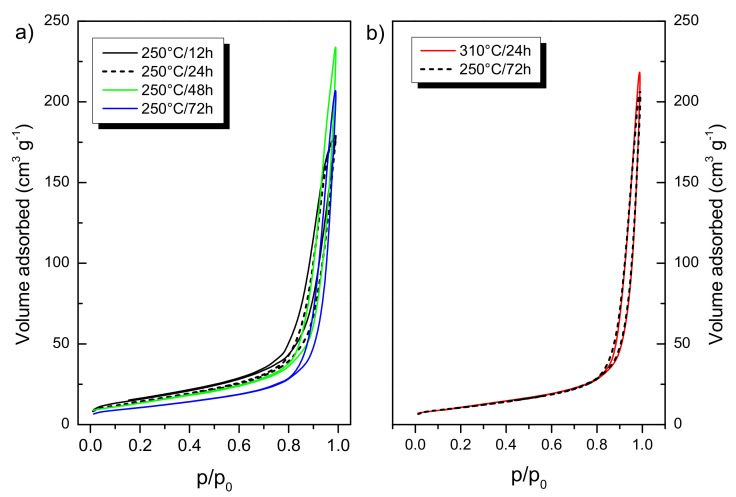
(**a**) N_2_-sorption isotherms of TiO_2_ NPs treated hydrothermally at 250 °C for 12 h, 24 h, 48 h, and 72 h; (**b**) shows the isotherms after hydrothermal treatment of TiO_2_ NPs at 250 °C for 72 h and 310 °C for 24 h.

As the crystallite size increased by the synthesis time, the specific surface area ($a_{BET}^{s}$) of the samples decreased (Table 1). The $a_{BET}^{s}$ values were 56, 51, 50, and 38 m^2^ g^−1^ for A12h, A24h, A48h, and A72h samples, respectively. A310C exhibited similar surface area (40 m^2^ g^−1^) to that of A72h, even though their synthesis conditions were significantly different. N_2_-sorption isotherms are depicted in Figure 2. Based on the International Union of Pure and Applied Chemistry (IUPAC) classification, all the isotherms can be classified to type II with H3 hysteresis loop, without indication of a plateau at high p/p_0_. These isotherms have been frequently obtained for metal oxide nanoparticles with substantial interstitial porosity [20]. A72h and A310C showed almost the same isotherms (Figure 2b). It can be concluded that the structural evolution of TiO_2_ NPs was significantly faster at 310 °C than 250 °C.

### 3.2. Morphology and Size Distribution of TiO_2_ NPs

**Figure 3:**
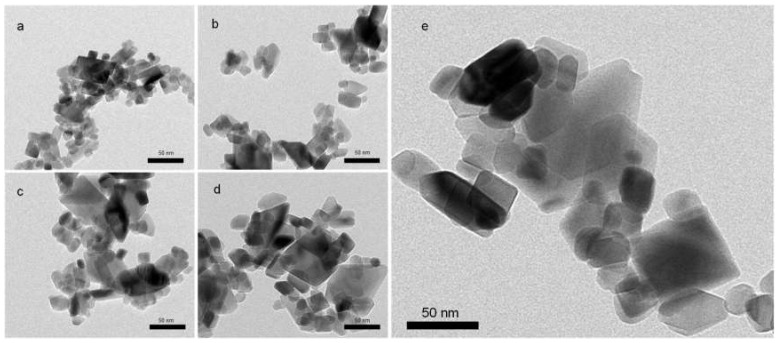
TEM images of TiO_2_ NPs treated hydrothermally at 250 °C for (**a**) 12 h, (**b**) 24 h, (**c**) 48 h, (**d**) 72 h and (**e**) at 310 °C for 24 h. Scale bars: 50 nm.

TEM images demonstrate that all the samples were composed of polydisperse TiO_2_ NPs with mixed morphology (Figure 3a–e). In good agreement with XRD results, larger particles were formed at prolonged synthesis time and at higher temperature. Sample A310C exhibited the largest TiO_2_ NPs (Figure 3e). The particle size varied over a wide range (~10−80 nm) for each sample. Particle size distribution cannot be evaluated accurately because of their overlap in the TEM images. TiO_2_ NPs with a size of ~10 nm were still present in the samples treated for longer time (72 h) or at 310 °C even though the relative amount of these tiny particles decreased at these conditions. Therefore, the size distribution became broader due to the formation of bigger particles at longer treatment times. Although the particle sizes depended on the synthesis duration, the morphology was very similar for all the samples. Both roughly spherical and polyhedral (faceted) nanoparticles were formed. The faceted nanoparticles exhibit truncated tetragonal bipyramidal geometry, which is characteristic for anatase nanocrystallites [22,23]. Elongated nanoparticles with rod-like morphology were also observed.

### 3.3. Optical Band Gap 

**Figure 4:**
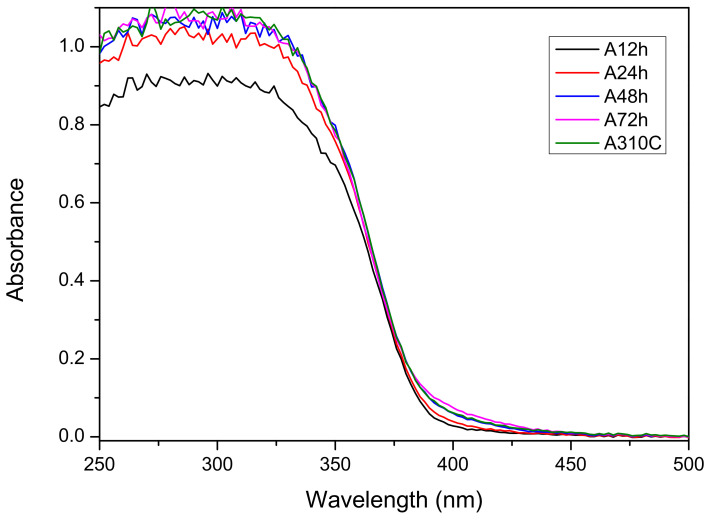
Diffuse reflectance UV-Vis spectra of TiO_2_ NPs treated hydrothermally for 12 h, 24 h, 48 h, and 72 h at 250 °C and at 310 °C for 24 h.

Diffuse reflectance ultraviolet-visible UV-Vis DR spectra were recorded for all samples (Figure 4). They show a similar absorption edge at ~387 nm. This coincides with a calculated band gap energy of ~3.2 eV, which is in good agreement with the value published previously for anatase [24,25]. However, with the increasing duration of the hydrothermal treatment, the absorption also becomes not negligible between 380 nm and 430 nm. Moreover, also in the case of sample A310C, this small but clear absorption is visible. Nevertheless, it is yet unclear what the underlying reason for the occurrence of this additional band is.

### 3.4. Chemical Composition at the Near-Surface Region

**Figure 5:**
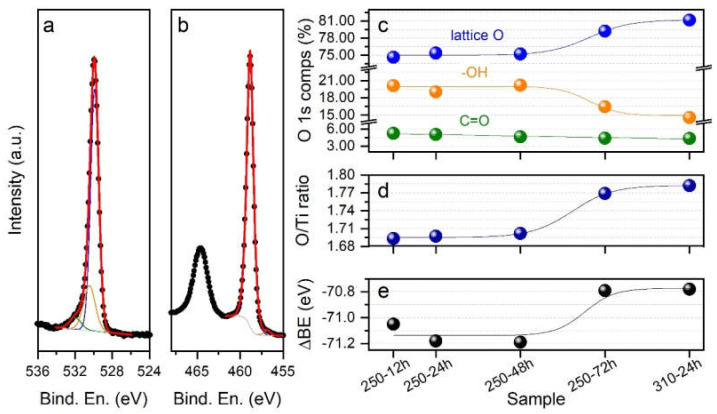
Results of the X-ray photoelectron spectroscopy (XPS) investigation. (**a**,**b**) XPS data collected over the O 1s and Ti 2p regions, respectively, on the A12h sample. The results of the best fitting procedure are also reported; (**c**) markers show the relative atomic concentrations of the O 1s components in the five analyzed samples, as obtained from the decomposition process. Lines are shown as a guide for the eye; (**d**) markers show the values of the O/Ti ratio, as obtained from the quantitative analysis of the XPS data. Here only the O 1s component assigned to lattice oxygen has been considered. The blue line is shown as a guide for the eye; (**e**) markers report the binding energy distance between the main O 1s component (i.e., lattice O) and the main Ti 2p peak (due to Ti(IV) species) for the five analyzed samples. The black line is shown as a guide for the eye.

The results obtained through XPS are summarized in Figure 5. Figure 5a,b shows the typical O 1s and Ti 2p spectra, respectively, in the case of the A12h sample. Similarly to what has already been reported in the literature, the O 1s spectrum (Figure 5a) can be decomposed into three components, centered at 529.9 ± 0.2 eV, 530.4 ± 0.2 eV and 531.9 ± 0.2 eV, that could be respectively attributed to crystal lattice oxygen (O-Ti), -OH and C=O groups adsorbed on the sample surface. The Ti 2p spectrum (Figure 5b) shows the presence of two peaks, namely Ti 2p_3/2_ and Ti 2p_1/2_, located at 458.8 ± 0.2 eV and 464.5 ± 0.2 eV, respectively. In agreement with previous results on related systems, we assigned the observed Ti signals to Ti(IV) species in TiO_2_. To obtain the best fitting of the data, we also included a Ti(III) component at (457.0 ± 0.2) eV; its weight, however, stayed below 2% of the overall Ti content, for all the investigated samples. The spectra collected on the other four samples (A24h, A48h, A72h, and A310C) do not differ significantly from those shown in Figure 5a,b, and are therefore not reported. The outcomes of the analysis of all the collected data are summarized in Figure 5c–e. In Figure 5c, the relative concentrations of the O 1s components are shown for the five investigated samples. The concentration of lattice O (blue markers), as well as that of -OH groups adsorbed at the sample surface (orange markers), is almost constant for samples treated at 250 °C for up to 48 h. In these samples, lattice O accounts for approximately 75% of the total oxygen content, while -OH groups represent approximately 20%. As the treatment duration was increased to 72 h, the lattice O content raised to approximately 79.5%, and the -OH group content decreased to approximately 16.5%. The A310C sample is characterized by the highest lattice O (81%) and by the lowest -OH groups (15%) contents. The concentration of C=O groups (green markers) is instead almost constant for all the samples in the 4.5−5% range. By calculating the area of the O and Ti peaks assigned to TiO_2_, we then calculated the O/Ti ratio for the analyzed samples. The results are plotted in Figure 5d; again, while the A12h, A24h, and A48h samples show similar values (O/Ti ~1.7), samples A72h and A310C are characterized by higher O/Ti values, approaching 1.8 in the case of the sample treated at 310 °C. For all the investigated samples, it is important to notice that a certain oxygen deficiency is present. The differences in stoichiometry are also somehow reflected in the binding energy distance between the main Ti 2p peak (due to Ti(IV) species) and the main O 1s component (i.e., lattice O), reported in Figure 5e. The higher the oxygen content is, the closer in energy the peaks are. It has to be noted that the reported energy distance for anatase or rutile crystalline TiO_2_ is in the order of −71.2 eV [26], in good agreement with what we obtained for the A24h and A48h samples. Considering that, according to XRD analysis, the A72h and A310C samples are those with a higher crystallinity, the observed shift might be an indication of, for example, a slight excess of positive charge on the surface Ti atoms (Ti^4+δ^) [27].

### 3.5. Photocatalytic Properties

**Figure 6:**
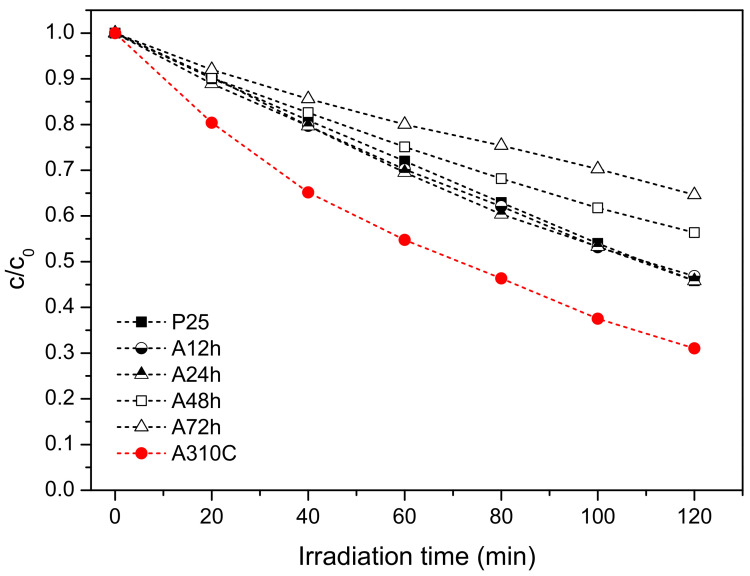
Photocatalytic degradation of phenol using different TiO_2_ NPs treated hydrothermally and P25 TiO_2_ (as reference photocatalyst) under UV-A light irradiation. The aqueous dispersions contained TiO_2_ NPs with a concentration of 0.5 mg/mL. The initial phenol concentration was 0.5 mM.

The photocatalytic activity of the samples was studied by photo-degradation of phenol, chosen as a model pollutant under UV irradiation. Since we applied a low-power (15 W) light source and the emitted radiation was of relatively low energy (UV-A), phenol degradation without photocatalysts was not detected during irradiation of 120 min. Besides, in the presence of TiO_2_ NPs but without application of UV light (dark experiments), phenol did not decompose. Figure 6 demonstrates that A310C has superior photocatalytic activity reaching ~70% phenol decomposition after 120 min. A12h, A24h, and commercial Degussa P25 show similar photoactivity and, by using these photocatalysts, ca. 55% of the initial phenol content was decomposed. The rate of photo-oxidation was significantly slower by using A48h and A72h, resulting ~44% and 36% efficiency for the phenol decomposition, respectively. Despite the similar crystallite size, specific surface area, and the chemical composition in the near-surface region, the photocatalytic activity of A310C and A72h differed considerably. 

### 3.6. Application of TiO_2_ NPs against Phytopathogens

Based on the phenol degradation experiments, the A310C photocatalyst was chosen for antibacterial tests. P25, which is a well-known commercial TiO_2_ with high photocatalytic activity, was also tested as reference photocatalyst. In the presence of UV-light, the living cell number did not decrease if bacteria were suspended only in physiological salt solution (0.9% NaCl solution). Furthermore, no antibacterial effect of TiO_2_ NPs was observed during the dark preincubation period (15 min), even when A310C or P25 TiO_2_ was applied (data not shown). 

**Figure 7:**
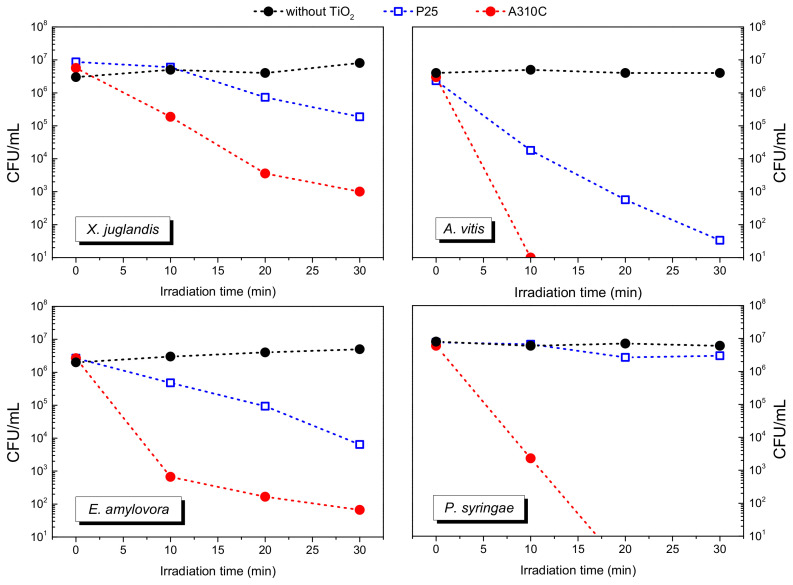
Antibacterial activity of A310C and P25 TiO_2_ photocatalysts against *Xanthomonas arboricola* pv. *juglandis*, *Allorhizobium vitis*, *Erwinia amylovora*, and *Pseudomonas syringae* under UV-A irradiation. The concentration of aqueous TiO_2_ dispersions was 0.5 mg/mL. Before UV-A irradiation, dispersions containing bacteria were kept in the dark for 15 min.

Under UV-irradiation, A310C showed a pronounced antimicrobial activity on all the investigated plant pathogenic bacteria. The kinetic curves (Figure 7) reveal that the order of susceptibility of tested bacteria using A310C is the following: *A. vitis* >> *E. amylovora* > *P. syringae* > *X. juglandis*. *A. vitis* showed the highest susceptibility towards TiO_2_ NPs, as the living bacterial cell number decreased by six orders of magnitude after 10 min irradiation. No viable cells of *A. vitis* could be detected after 20 min UV-irradiation. *X. juglandis* exhibited the lowest susceptibility to UV-irradiated A310C. Our results demonstrate that the antibacterial efficacy of A310C was significantly higher than that of P25. The reference P25 TiO_2_ was not able to completely eliminate the living bacteria in any of the investigated species within the applied time frame. The order in the susceptibility for P25 TiO_2_ was different: *A. vitis* >> *E. amylovora* > *X. juglandis* > *P. syringae.* For *A. vitis,* viable bacterial cells decreased more than four orders of magnitude in P25 TiO_2_ suspension after 30 min UV irradiation. In the case of *X. juglandis* and *E. amylovora* these values were around 1.5 and 2.5, respectively, after 30 min and only a minimal antibacterial effect was detected in the case of *P. syringae*.

### 3.7. Main Reactive Oxygen Species-Formation Kinetics of OH^●^ and O_2_^●−^ Radicals

**Figure 8:**
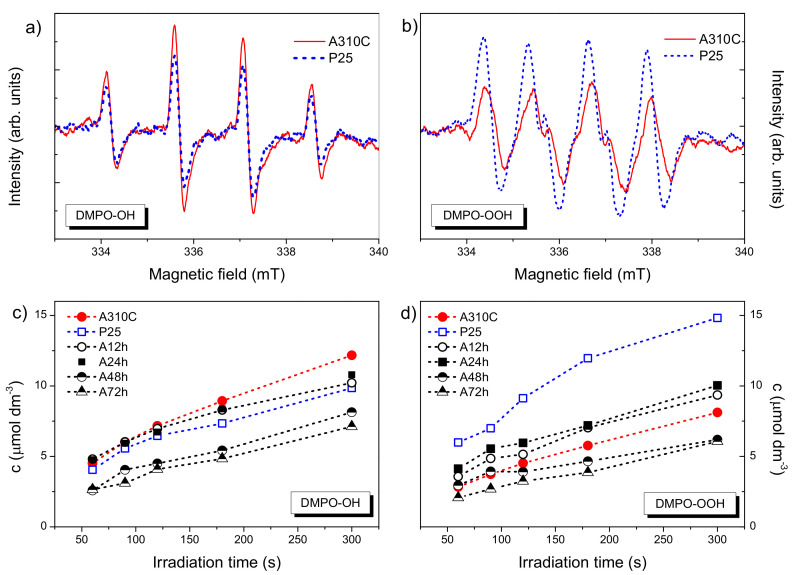
Results of the electron paramagnetic resonance (EPR) spectroscopy investigation. (**a**,**b**) EPR spectra of DMPO-OH and DMPO-OOH adducts recorded after UV light irradiation for 300 s using A310C and Degussa P25 photocatalysts; (**c**,**d**) formation of DMPO-OH and DMPO-OOH adducts in different TiO_2_ dispersions as a function of UV irradiation time. The dispersions contained 0.1 mg/mL of TiO_2_ photocatalysts.

The ability of TiO_2_ NPs to produce hydroxyl (OH^●^) or superoxide radicals (O_2_^●−^) upon UV photoexcitation was studied via an EPR technique using DMPO as a spin trapping agent. In aqueous TiO_2_ NPs dispersion exposed to UV light, a typical 1:2:2:1 four-line EPR signal of DMPO-OH (2-hydroxy-5,5-dimethyl-1-pyrrolidinyloxy) with hyperfine splitting constants a_N_ = a_H_ = 1.49 mT was recorded (Figure 8a). Nevertheless, the formation of DMPO-OOH was not observed in aqueous medium because the spin adduct formation is kinetically slower than the protonation of O_2_^●−^ followed by the disproportion of the forming hydroperoxyl radical (HO_2_^●^) [28]. Thus, for the detection of O_2_^●−^, instead of water, aerated DMSO was chosen as a reaction medium, considering that DMSO is able to stabilize O_2_^●−^ [29]. The obtained EPR spectrum of DMPO-OOH (hyperfine constants a_N_ = 1.37 mT, a_Hβ_ = 1.0 mT) is presented in Figure 8b. At the same reaction condition, the signal intensity of DMPO-OH was higher for A310C than P25 (Figure 8a). In contrast, Degussa P25 produced more O_2_^●−^ radicals as revealed via the comparison of DMPO-OOH spectra displayed in Figure 8b. The kinetic curves for the formation of DMPO-OH and DMPO-OOH are presented in Figure 8c,d. The OH^●^ formation was more intense than that of O_2_^●−^ formation for all TiO_2_ NPs treated hydrothermally. The level of DMPO-OH was the highest using A310C followed by A24h, A12h, and P25. The concentration of DMPO-OH and DMPO-OOH were the lowest by using A48h and A72h, showing long-term treatment had an adverse effect on the formation of the OH^●^ and the O_2_^●−^. Interestingly, P25 showed the highest activity for the formation of O_2_^●−^. 

## 4. Discussion

In the devised two-step wet-chemical synthesis, the first step was the hydrolysis of the titanium isopropoxide resulting in hydrous titania (TiO_2_· *n*H_2_O). In the second step, the obtained material was treated hydrothermally. Our method was performed without using surfactant or doping agent leading to high-purity TiO_2_ as confirmed by XPS measurements. Since the reaction product obtained in the first step is amorphous, and therefore almost photo-catalytically inactive (data not shown), the crystallization of the samples is required. Hydrothermal synthesis is an ideal method to obtain metal oxides with high crystallinity under relatively low temperature and elevated pressure [20]. For TiO_2_ NPs fabrication, hydrothermal synthesis at 250 °C or higher temperature (e.g., 310 °C) is rarely applied which is probably due to the fact that these conditions required pressure-tight reaction vessels. Consequently, the available information about the structural and photocatalytic properties of these nanomaterials is limited in the literature. Our results show that particle formation from the amorphous precursor (TiO_2_· *n*H_2_O) can be adjusted over the reaction temperature and duration of the treatment (Table 1). The average crystallite size was controlled between ~30 and 50 nm applying 12–72 h treatment at 250 °C. Anatase nanocrystallites with 52.2 nm average size formed at 310 °C for 24 h. It can be stated that either the duration of the treatment or the higher temperature favored the formation of the larger nanocrystallites. Furthermore, the crystallinity of the samples can be enhanced by the hydrothermal treatment (Table 1). After 12 h treatment time, relatively high crystallinity (~75%) has already been achieved. Longer treatment time (24–72 h) resulted in further improvement in the crystallinity but the increment was slower in this period. The crystallization was faster at 310 °C. At this temperature, ~95% crystallinity was achieved after 24 h. The applied conditions favored the anatase crystal phase formation (Figure 1). Enhanced crystallinity can improve the photocatalytic activity [21,30]. The recombination probability of photogenerated charge carriers is lower for the more crystalline samples [20], while high concentrations of structural defects of amorphous TiO_2_ form recombination centers [31]. Anatase formation has an advantage because both rutile and brookite generally show lower photocatalytic activity [32,33]. Only A72h was composed of a significant amount of rutile (7.6%) which can form from either amorphous titania or from anatase crystal phase by recrystallization (Figure 1). A trace of brookite was also detected in A12h (Figure 1). Since the samples were non-porous and only interstitial porosity was observed (Figure 2) the specific surface area correlated inversely with the average crystallite size determined via the Scherrer equation (Table 1). According to this relation, higher temperature or prolonged reaction time of the hydrothermal treatment induced the lower specific surface area of the samples. Although the particle size increased during the hydrothermal treatment, very similar morphology was observed for each sample (Figure 3). Furthermore, optical properties did not differ significantly exhibiting 3.2 eV band gap energies for all samples (Figure 4). This energy was supplied without difficulty by the UV-A light source used in the experiments (Figure S1).

For the investigation of the activity of a photocatalyst two main parameters, crystallite size and crystallinity are generally considered. It was reported that photo-reactivity of anatase nanoparticles synthesized under supercritical water-isopropanol conditions increased with increasing crystallite size but was independent of the crystallinity [34]. For our samples, since crystal size and crystallinity varied in parallel with the treatment time (Table 1), it was not possible to determine which parameter is primarily responsible for the enhanced photocatalytic activity. Nevertheless, the sample (A310C) with the largest crystallite size showed the highest photoactivity, which is in good agreement that reported by Wang et al. [34]. A310C possessed the lowest specific surface area indicating that this parameter is not a critical factor to obtain titania with high photocatalytic activity. However, it is also clear that not only crystal size or crystallinity play key roles. This can be easily conceived by comparing the structural properties, and photocatalytic activity of our samples. The crystallinity and crystallite size of titania samples increased in parallel with the prolonged treatment time (Table 1). While these structural changes pointed to one direction as a function of treatment time, photocatalytic activity increased up to 24 h, and then significantly decreased (Figure 6). A24h (average crystallite size (*d* = 36.7 nm, crystallinity = 86.7%) and A48h (*d* = 37.7 nm, crystallinity = 87.2%) with nearly the same crystallite size and crystallinity showed significant differences in both phenol degradation and production of ROS (Figure 6 and Figure 8). From these observations we can conclude that even though the crystallinity/crystallite size plays a role, another parameter seems to determine the activity much more, creating differences in the rate of ROS formation. This hypothesis is further supported by the fact that A72h (*d* = 48.7 nm, crystallinity = 99.6%) and A310C (*d* = 52.2 nm, crystallinity = 94.8%) also showed similar structural properties with the highest difference in photocatalytic activity. The near-surface composition, therefore, was also checked (Figure 5). Surface OH groups can play an important role in the charge trapping processes and the enhancement of photocatalytic activity [35,36]. Bridge OH groups, on the one hand, react with positive electron holes [37], reducing the rate of electron and hole recombination’s. On the other hand, very reactive hydroxyl radicals are formed in the reaction, which can easily react with organic compounds. However, XPS study did not confirm significant differences in OH coverage between A72h and A310C samples (Figure 5c). Despite the similarity in the surface composition of these samples, the difference for the efficiency of phenol degradation was ~35%. Consequently, further studies are necessary to clarify the reason for their high photoactivity. Nevertheless, our results show that hydrothermal treatment at high temperature and short times favored the formation of titania photocatalyst with high activity.

For testing the antibacterial activities of P25 and A310C (Figure 7), four plant pathogenic bacteria were chosen, with the common feature being that they infect the sun-exposed aboveground parts of the target plants, like leaves, buds, fruit, or young twigs. *Erwinia amylovora* is the causative agent of fireblight, a serious disease of some *Rosaceae* plants, including apple, pear, et cetera [38]. *Xanthomonas arboricola* pv. *juglandis* is one of the most prevalent infective diseases of walnut that causes walnut blight [39]. *Allorhizobium vitis* (formerly *Agrobacterium vitis*) is one of the most serious bacterial pathogens of grapevine causing the crown gall disease [40]. *Pseudomonas syringae* has the capacity to infect a wide range of species and causes shoot and flower blights, canker, and diebacks [41]. Since these sites of the infections are sun-exposed parts of the plants, antimicrobial photocatalysts are potent candidates for prevention and treatment. The reason of the superior antibacterial activity of A310C compared to P25 can be deduced from its enhanced and unique ROS production pattern that was characterized with increased OH^●^ radical production in the EPR measurements (Figure 8). OH^●^ is the most reactive ROS. While the elimination of the two other ROS-like H_2_O_2_ and O_2_^●−^ from around the bacterial cell can be performed by protective enzyme-based systems (SodA, SodB, SodC, AhpCF, KatG, KatE) [42], protection or detoxification of OH^●^ can only be performed by certain radical scavengers [43]. Recently, sugars have become more widely recognized as members of the non-enzymatic antioxidant family in plants and yeasts [44,45] as the outer surface of bacterial cells are also covered by large amounts of sugars. This exopolysaccharide layer was reasonably thought to serve as a protective sheath around the bacterial cell. The relatively high rate of differences among the susceptibilities of the investigated four plant pathogenic species could be rooted in the variation of thickness, sugar composition, degree of phosphorylation, and presence of other modifications of the lipopolysaccharide (LPS) structures as its composition is strongly species and genus dependent, and have important implications for interactions with the bacterium’s environment [46]. A limited number of reports have earlier analyzed the role of these exopolysaccharide structures in the plant defense against reactive oxygen species in the case of the four bacterium species and documented the vital importance of ROS balance during pathogenesis [47,48,49].

The different susceptibilities among the investigated species could be explained by genus- or species-dependent properties. How the bacterial cell envelope is protected from oxidative damage is much less understood since bacterial defense mechanisms against oxidative stresses have been extensively studied only on cytoplasmic levels [42]. The nature of the ROS and the repertoire of defense mechanisms could determine the survival of a bacterium, that could be demonstrated in case of *P. syringae* and *A. vitis*, as both of them were very sensitive to A310C, but in contrast to *A. vitis*, *P. syringae* was resistant to P25. Presently we can only hypothesize that *A. vitis* possesses a more limited enzymatic defense mechanism against O_2_^•−^ radicals that leads to a sensitive phenotype against P25. How exactly sugar- and chain length variability, species-dependent membrane protein composition, and enzyme-based defense mechanisms of these bacteria influence the susceptibility against ROS requires further studies with isogenic mutants.

To sum up, we synthesized TiO_2_ NPs with very high photo-catalytic activity by using a simple hydrothermal method. It was found that the obtained TiO_2_ NPs produce OH^●^ radicals effectively, and consequently, they proved to be effective for the inactivation of plant pathogens bacteria. Although our in vitro experiments are very promising for the plant protection applications, further studies are necessary to examine the phytotoxicity of TiO_2_ NPs. ROS as signaling molecules, can definitely influence several physiological processes in plants [50,51,52,53]. If plant defense adequately alleviates the ROS-induced oxidative processes, plant protection via photo-reactive TiO_2_ NPs can be successful. This essentially requires that, under the desired conditions, the elevated ROS level can be tolerated by the plants without severe damages, while the TiO_2_-induced ROS should kill the pathogens. For the eliminating of ROS, plants use both enzymatic and non-enzymatic antioxidants [54,55]. In grapevines, for example, flavonol glycosides play a crucial role in the plant’s defense [24]. Recently, we found that these compounds are potent scavengers of OH^●^ radicals [56].

## 5. Conclusions

Nanocrystalline TiO_2_ photocatalysts were successfully prepared by a wet-chemical method in which structural and photocatalytic performance can be tuned by the applied hydrothermal treatment. The prolonged duration or higher temperature of the hydrothermal treatment led to an increase in crystallinity and crystal size while decreasing the specific surface area of TiO_2_ NPs. By increasing the duration of the hydrothermal treatment at 250 °C, the photocatalytic activity increased up to 24 h and then significantly decreased. Shorter treatment (24 h) at higher temperature (310 °C) resulted in a more active photocatalyst. For the efficient photo-catalytic ROS formation, the crystallinity/crystallite size of TiO_2_ NPs might play an important role since amorphous TiO_2_ is inactive. However, our results show that not only crystallinity, and chemical composition of the near-surface region are of crucial importance, but other parameters, not visible here in the studied properties have a much more pronounced impact on ROS formation and photoactivity. The results also showed that BET surface area was not a dominant factor. In addition, EPR measurements revealed that UV-A-irradiated anatase nanocrystallites produced relatively more OH^●^ than O_2_^•−^. The formation of these very reactive OH^●^ determined the photo-catalytic activity of the TiO_2_ NPs. In vitro application, the plant pathogens showed different susceptibilities to the TiO_2_ NPs but all bacterium species were inactivated rapidly under UV-A irradiation in the presence of the TiO_2_ NPs. These results indicate that anatase TiO_2_ nanocrystallites are very promising in the photo-catalytic-based inactivation of plant pathogens. However, further studies are required to reveal the phytotoxicity of TiO_2_ NPs. ROS induced by photo-catalytically active TiO_2_ NPs are an effective weapon against pathogens but high levels of them can lead to serious damages in plant tissues via oxidative processes.

## Figures and Tables

**Table 1:** Crystal phase composition and average crystallite size of anatase and BET surface areas for TiO_2_ NPs treated hydrothermally.

Sample ID	Anatase (wt %)	Rutile (wt %)	Brookite (wt %)	Crystallinity (wt %)	Crystallite Size ^a^ (nm)	Specific Surface Area ^b^ (m^2^ g^−1^)
A12h	75.5	nd	trace	75.5	29.7	56
A24h	86.1	0.6	nd	86.7	36.6	51
A48h	88.2	nd	nd	88.2	37.6	50
A72h	92.4	7.6	nd	99.6	48.7	38
A310C	94.1	0.7	nd	94.8	52.2	40

^a^ Average crystallite size (*d*) was estimated from the full-width at half maximum of the d_101_ peak of anatase using the Scherrer equation; ^b^ Specific surface area was calculated with the Brunauer–Emmett–Teller (BET) equation using the data of N_2_-sorption measurements; nd: not detected.

## Supplementary Materials

The following are available online at https://www.mdpi.com/2079-4991/10/9/1730/s1, Figure S1: UV-Vis spectrum of the light source used for the experiments.
