# Supplementary material for: Photocatalytic Inactivation of Plant Pathogenic Bacteria Using TiO2 Nanoparticles Prepared Hydrothermally
Source: Nanomaterials (Basel). 2020 Aug 31;10(9):1730. doi: 10.3390/nano10091730 (PMC7558638; doi:10.3390/nano10091730)
Supplement: Supplementary file 1 [file nanomaterials-10-01730-s001.pdf]

nanomaterials

Supplementary Materials:

Photocatalytic Inactivation of Plant Pathogenic Bacteria Using TiO_2 Nanoparticles Prepared Hydrothermally

László Kőrösi [1,*], Botond Pertics [2], György Schneider [2], Balázs Bognár [3], János Kovács [4], Vera Meynen [5], Alice Scarpellini [6], Lea Pasquale [7] and Mirko Prato [7]

[1] Research Institute for Viticulture and Oenology, University of Pécs, Pázmány P. u. 4, H-7634 Pécs, Hungary
[2] Department of Medical Microbiology and Immunology, Medical School, University of Pécs, Szigeti st. 12, H-7624 Pécs, Hungary; pertics.botond@pte.hu (B.P.); schneider.gyorgy@pte.hu (G.S.)
[3] Institute of Organic and Medicinal Chemistry, University of Pécs, Szigeti st. 12, H-7624 Pécs, Hungary; balazs.bognar@aok.pte.hu
[4] Environmental Analytical and Geoanalytical Research Group, Szentágothai Research Centre, University of Pécs, Ifjúság u. 20, H-7624 Pécs, Hungary; jones@gamma.ttk.pte.hu
[5] Laboratory of Adsorption and Catalysis, Department of Chemistry, University of Antwerp, Universiteitsplein 1, 2610 Wilrijk, Belgium; vera.meynen@uantwerpen.be
[6] Electron Microscopy Facility, Istituto Italiano di Tecnologia, via Morego 30, 16163 Genova, Italy; alice.scarpellini@iit.it
[7] Materials Characterization Facility, Istituto Italiano di Tecnologia, via Morego 30, 16163 Genova, Italy; Lea.Pasquale@iit.it (L.P.); Mirko.Prato@iit.it (M.P.)
* Correspondence: korosi.laszlo@pte.hu; Tel.: +36-(72)-517933

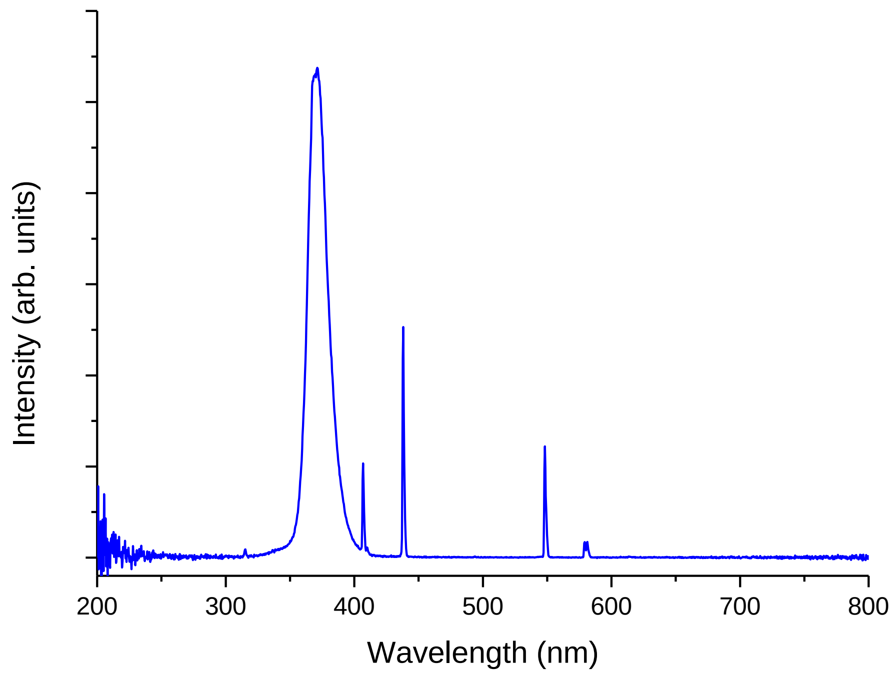

Figure S1. UV-Vis spectrum of Blacklight 368 F15W/T8/BL368 UVA lamp (Sylvania) used for the experiments.
